# BMC Medical Ethics reviewer acknowledgement 2014

**DOI:** 10.1186/1472-6939-16-5

**Published:** 2015-02-10

**Authors:** Clare Partridge

**Affiliations:** BioMed Central, Floor 6, 236 Gray’s Inn Road, London, WC1X 8HB UK

## Abstract

The editors of BMC Medical Ethics would like to thank all our reviewers who have contributed to the journal in Volume 15 (2014).

**Aslihan Akpinar**

Turkey

**Eman Al Gaai**

Saudi Arabia

**Amel Alghrani**

UK

**Afshan Ali**

Saudi Arabia

**M. Z. Alkawi**

Saudi Arabia

**James A Anderson**

Canada

**Melissa Anderson**

USA

**Chike Anibeze**

Nigeria

**Marco Annoni**

Italy

**Paul Appelbaum**

USA

**Julie Armin**

USA

**Atsushi Asai**

Japan

**Richard Ashcroft**

UK

**Carol Ashton**

USA

**Peter John Aspinall**

UK

**Jacquineau Azetsop**

Chad

**Francis Barchi**

USA

**Lianne Barnieh**

Canada

**Drue Barrett**

USA

**Philip Bejon**

Kenya

**Solomon (Solly) Benatar**

South Africa

**Sara Bergstresser**

USA

**Guido Biasco**

Italy

**Ariella Binik**

UK

**Giles Birchley**

UK

**Felicity Bishop**

UK

**Charlyn Black**

Canada

**Isra Black**

UK

**Fiona Boland**

Ireland

**Roseanna Bourke**

New Zealand

**Deborah Bowen**

USA

**Marek Brabec**

Czech Republic

**Iain Brassington**

UK

**Gina Bravo**

Canada

**Niamh Brennan**

Ireland

**Howard Brody**

USA

**Kyle Brothers**

USA

**Hilde Buiting**

Netherlands

**Eric Campbell**

USA

**Krysia Canvin**

UK

**Adriana Ceci**

Italy

**Kenneth Chambaere**

Belgium

**Audrey Chapman**

USA

**Kath Checkland**

UK

**Lei-Shih Chen**

USA

**Gabrielle Christenhusz**

Belgium

**Murat Civaner**

Turkey

**Ellen Clayton**

USA

**Adam Cohen**

Netherlands

**Joachim Cohen**

Belgium

**Luana Colloca**

USA

**James Colquitt**

Armenia

**Andrew Courtwright**

USA

**John Coverdale**

USA

**Andrew Crowden**

Australia

**Rafael Dal-Ré**

Spain

**Bege Dauda**

Belgium

**Angus Dawson**

UK

**Liza Dawson**

USA

**Jacques Demotes**

France

**Ioannis Dimoliatis**

Greece

**Edward Dove**

Canada

**Anne-Elisabeth Driessen**

Netherlands

**Brian Earp**

UK

**Marleen Eijkholt**

USA

**Susan Ellenberg**

USA

**Nathan Emmerich**

UK

**Miran Epstein**

UK

**Daniele Fanelli**

UK

**Conrad Fernandez**

Canada

**Joel Frader**

USA

**Samuel Frank**

USA

**Lucy Frith**

UK

**Davina Ghersi**

Australia

**Nader Ghotbi**

Japan

**Noor Giesbertz**

Netherlands

**Simona Giordano**

UK

**Alberto Giubilini**

Australia

**Aaron Goldenberg**

USA

**Joseph Graves Jr.**

USA

**Kalle Grill**

Sweden

**Thomas Grisso**

USA

**Ronella Grootens-Wiegers**

Netherlands

**Marilys Guillemin**

Australia

**Tolga Guven**

Turkey

**Bridget Haire**

Australia

**Arja Halkoaho**

Finland

**Muhammad Hammami**

Saudi Arabia

**Sumaya Hammami**

USA

**Martyn Hammersley**

UK

**Wolfgang Hampe**

Germany

**Sven Ove Hansson**

Sweden

**Gert Helgesson**

Sweden

**Kristien Hens**

Netherlands

**Jonathan Herring**

UK

**Spencer Hey**

Canada

**Soren Holm**

UK

**Neil A. Holtzman**

USA

**Matthew Hotopf**

UK

**Heidi C Howard**

Sweden

**Sara Hull**

USA

**Matthew Hunt**

Canada

**Richard Huxtable**

UK

**Rick Iedema**

Australia

**Carel Ijsselmuiden**

Switzerland

**Julia Inthorn**

Germany

**Saima Iqbal**

Pakistan

**Jonathan Ives**

UK

**Simon Jenkins**

UK

**Steven Joffe**

USA

**Yann Joly**

Canada

**Karsten Juhl Jørgensen**

Denmark

**Ralf Jox**

Germany

**Niklas Juth**

Sweden

**Gernot Kaiser**

Germany

**Dorcas Kamuya**

Kenya

**Hitomi Kataoka**

Japan

**Georg Kemmler**

Austria

**Melissa Kendall**

Australia

**Jack Kennelly**

New Zealand

**Ian Kerridge**

Australia

**Sy Kim**

USA

**Sharon Kling**

South Africa

**Sheldon Krimsky**

USA

**Lisa Lee**

USA

**Simon J. Craddock Lee**

USA

**Penney Lewis**

UK

**Charles Lidz**

USA

**Geoffrey Lomax**

USA

**Jose-Antonio Lopez-Pina**

Spain

**Sana Loue**

USA

**Jayne Lucke**

Australia

**Tamra Lysaght**

Singapore

**Kristin Bakke Lysdahl**

Norway

**Graeme Maclaren**

Singapore

**Shoichi Maeda**

Japan

**Morten Magelssen**

Norway

**Nicola Magnavita**

Italy

**Aisha Malik**

UK

**Phillipa Malpas**

New Zealand

**Vicki Marsh**

Kenya

**Deborah Mascalzoni**

Italy

**Bojana Matejic**

Serbia

**Sheryl McCurdy**

USA

**Martin McKneally**

Canada

**Robert McMaster**

UK

**Lindsay McNair**

USA

**Darlene McNaughton**

Australia

**Andrew McRae**

Canada

**Marcel Mertz**

Germany

**Kenneth Miller**

USA

**Francesca Minerva**

Australia

**Bert Molewijk**

Netherlands

**Sassy Molyneux**

Kenya

**Keymanthri Moodley**

South Africa

**Paul Mueller**

USA

**Paul Ndebele**

Zimbabwe

**Peter A Newman**

Canada

**Dianne Nicol**

Australia

**Erik Nord**

Norway

**Susan L Norris**

USA

**David Obree**

UK

**Elke Birgit Ochsmann**

Germany

**Anke Oerlemans**

Netherlands

**Sadayoshi Ohbu**

Japan

**Kanu Okike**

USA

**Taketoshi Okita**

Japan

**Stuart Oultram**

UK

**John Owens**

UK

**Vural Ozdemir**

Canada

**Bennett Pafford**

USA

**Vassilios Papalois**

UK

**Michael Parker**

UK

**John Porter**

UK

**Corinna Porteri**

Italy

**Rouven Christian Porz**

Switzerland

**Stefan Priebe**

UK

**Marta Pulido**

Spain

**Divya Rajaraman**

India

**Flavia Ramos**

Brazil

**Stella Reiter-Theil**

Switzerland

**Emmanuelle Rial-Sebbag**

France

**Christopher Robertson**

USA

**Eduardo Rodriguez**

Chile

**Wendy Rogers**

Australia

**Lainie Ross**

USA

**Theresa Rossouw**

South Africa

**Mark Rothstein**

USA

**Michael Rowe**

USA

**Sabine Salloch**

Germany

**Lars Sandman**

Sweden

**Pamela Sankar**

USA

**Julian Savulescu**

UK

**Jerome Schofferman**

USA

**Sarah Hudson Scholle**

USA

**Vera Scott**

South Africa

**Clive Seale**

UK

**Janet Seeley**

Uganda

**Mary Seeman**

Canada

**Nayha Sethi**

UK

**Jane Seymour**

UK

**Julian Sheather**

UK

**Diego Silva**

Canada

**Jerome Singh**

South Africa

**Catherine Slack**

South Africa

**Elpidoforos Soteriades**

Cyprus

**Jocelyne St-Arnaud**

Canada

**Ruth Stirton**

UK

**Erwin Stolz**

Austria

**Daniel Strech**

Germany

**Mia Svantesson**

Sweden

**Elisabeth Svensson**

Sweden

**Leslie Swartz**

South Africa

**Emmanouil Symvoulakis**

Greece

**Anne-Marie Tassé**

Canada

**Patrick Taylor**

USA

**Sally Theobald**

UK

**Engelbert Theurl**

Austria

**Paulina Tindana**

Ghana

**Gustav Tinghög**

Sweden

**Tsuen-Chiuan Tsai**

Taiwan

**Kristen Underhill**

USA

**Johannes Van Delden**

Netherlands

**Rieke Van Der Graaf**

Netherlands

**Joseph Verheijde**

USA

**A.M. Viens**

UK

**Julia Wade**

UK

**Elizabeth Wager**

UK

**Rebecca Walker**

USA

**James Warner**

UK

**Douglas Wassenaar**

South Africa

**Robert Wheeler**

UK

**Karolyn White**

Australia

**David Kenneth Whynes**

UK

**Guy Widdershoven**

Netherlands

**Garrath Williams**

UK

**Bryn Williams-Jones**

Canada

**Erica Witkamp**

Netherlands

**Amanda Wolf**

New Zealand

**Luke Wolfenden**

Australia

**Heiner Wolters**

Germany

**Simon Woods**

UK

**Christina Zampas**

Sweden

**Ma'N H. Zawati**

Canada

**Deborah Zion**

Australia

**Daniella Zipkin**

USA

**Matjaz Zwitter**

Slovenia

